# Cutaneous Metastasis in the Setting of Both Colon and Breast Primary Malignancies

**DOI:** 10.1155/2020/8852459

**Published:** 2020-09-29

**Authors:** Mary Junak, Hunter Jecius, Jennifer Erdrich

**Affiliations:** Institutional Mailing Address, University of Arizona, Department of Surgery, Tucson, AZ 85724, USA

## Abstract

Colorectal cancer (CRC) is the third most diagnosed cancer in the United States, and many patients unfortunately have metastases at the time of their diagnosis. Cutaneous metastases of CRC have been reported in few journals and primarily as case reports due to their rarity. Here, we present the case of an 83-year-old woman with recently resected colon cancer, T4aN1bMx stage IIIB. She presented to our clinic for evaluation of a right midback mass, and a punch biopsy revealed dermal involvement by invasive, poorly differentiated carcinoma with epidermoid features. The mass was excised, and we ordered a PET scan in search of the primary tumor, which at that time was suspected to be of skin cancer origin. Surprisingly, this revealed a second malignancy triple-negative invasive ductal carcinoma of the left breast. The back mass stained positive for CK20, which was compatible with a metastasis from a colonic primary. After initially declining adjuvant therapy, the patient completed one cycle of capecitabine and oxaliplatin, which she tolerated poorly. She continued to further decline, developed widespread cutaneous metastases, and went home on hospice. Cutaneous lesions are an exceedingly rare site of metastasis for colon adenocarcinoma, and their clinical presentation can vary widely. It is important for providers to investigate any new skin lesion in a patient with a recent or remote history of malignancy, even if there were no sites of distant metastasis at initial diagnosis.

## 1. Introduction

Colorectal cancer (CRC) is the fourth leading cause of cancer-related deaths worldwide and the third most diagnosed cancer in the United States [1]. Overall incidence of CRC has decreased in recent decades secondary to implementation of effective screening guidelines and early detection of primary malignancies. However, most patients diagnosed with CRC die from the malignancy itself, and the National Institutes of Health Surveillance, Epidemiology, and End Results Program (SEER) estimates over 51,000 deaths in 2019 due to CRC alone [2, 3]. Development of metastatic disease largely contributes to this figure, and many patients unfortunately have metastases at the time of their diagnosis [4, 5]. The liver is the most common site of CRC metastases presumably due to venous blood flow, but recent studies suggest that anatomic location and histological subtype significantly affect the pattern of metastases when considering organs beyond the liver. The most common nonhepatic colorectal metastases are to the thorax, nervous system, skeletal system, and peritoneum [6]. Brain, ovary, adrenal, and skin are rare sites of metastases, and the mechanisms of these progressions are not fully understood. Cutaneous metastasis arising from internal malignancy is extremely rare occurring in 0.001% of all skin biopsies performed [7]. Metastatic cutaneous manifestations have been reported to occur in 0.7–9% of patients with visceral neoplasms, with cutaneous metastases of CRC having a prevalence of 5.8% and signaling advanced disease [8–12]. Cutaneous metastases of CRC have been reported in few journals and primarily as case reports due to their rarity. More often, cases of breast cancer followed by lung, colorectal, renal, ovarian, and bladder cancers involve discussions of cutaneous metastases. These present heterogeneously as slow-growing tumors to rapidly growing dermal or subcutaneous nodules with either intact or ulcerated epidermis [13]. We present a unique case of a patient with a past medical history of basal and squamous cell carcinoma who presented with what was ultimately found to be cutaneous metastases from a colon primary without hepatic involvement in the context of a new synchronous diagnosis of an invasive ductal carcinoma of the breast.

## 2. Case Presentation

**Figure 1:**
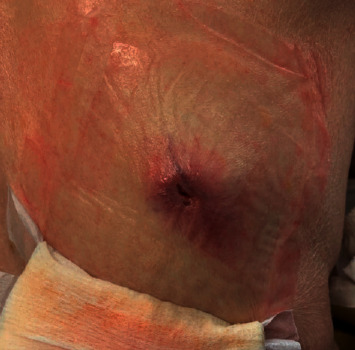
Right midback, nonpigmented, raised mass with the central area of erythema and serous drainage.

**Figure 2:**
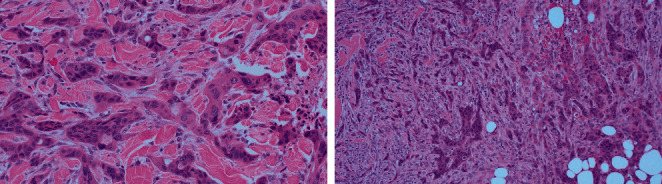
Microphotographs of the excisional biopsy of the skin lesion showing infiltrating cords and solid nests of epithelial cells within the deep dermis (H&E, 20x and 40x, respectively).

An 83-year-old woman with a history of basal cell carcinoma and squamous cell carcinoma presented to our clinic for evaluation of a right midback mass. She initially presented to her dermatologist with what appeared to be an inflamed sebaceous cyst, but after a failed trial of Keflex, a punch biopsy performed at an outside institution revealed dermal involvement by invasive, poorly differentiated carcinoma with epidermoid features. By immunohistochemistry, the tumor cells expressed high-molecular weight keratin, BerEP4, and EMA but were negative for P63, GCDFP15, estrogen receptor, progesterone receptor, TTF-1, CEA, cytokeratin 7, and cytokeratin 20. At this point, the etiology of her mass was unclear; however, it should be noted that the patient was 6 weeks post-op from a laparoscopic-assisted left colectomy at an outside institution for an obstructing, poorly differentiated colon adenocarcinoma with squamous differentiation and 3/25 lymph nodes positive for metastatic carcinoma (pT4aN1b). She declined chemotherapy. Her physical exam on presentation was significant for a 4 × 4 cm firm, nonpigmented mass with a centrally raised 2 × 3 cm area of erythema surrounding a punctate opening from the recent biopsy with serous drainage (Figure 1). We ordered a PET scan prior to the excision in order to identify the primary tumor. PET scan was remarkable for a large intensely FDG-avid cutaneous/subcutaneous malignant mass in the right midback with FDG-avid right axillary nodal involvement, focal FDG avidity at the left colon anastomotic site, an intensely FDG-avid left peritoneal node in the left lower quadrant adjacent to the surgical bed suspicious for nodal involvement from the colon primary, and an FDG-avid left lateral breast hyperdense mass. The patient subsequently underwent a wide local excision of the cutaneous mass on her back with 1 cm margins and a right axillary sentinel lymph node biopsy in which 3 lymph nodes were removed. Pathology report showed poorly differentiated carcinoma with squamous differentiation, negative margins, and 1/3 lymph nodes positive. Histologically, infiltrating cords and solid nests of malignant epithelial cells were noted (Figure 2).

The patient underwent additional workup for the PET findings. The patient returned to the clinic and reported that her last mammogram was performed over one year ago but was negative for malignancy. She denied breast skin changes, nipple discharge, and noticeable breast masses. Physical exam revealed a round, mobile palpable mass at 1 : 00 approximately 5 cm from the left nipple-areola complex. While the breast mass appeared benign, a bilateral diagnostic mammogram and left breast ultrasound were ordered to rule out malignancy in light of her current presentation of carcinoma with unknown origin. Mammography confirmed a suspicious mass in the left breast, and pathology from the ultrasound-guided biopsy revealed invasive ductal carcinoma, grade 3 (ER 0%, PR 0%, HER-2 1+, Ki-67 80%). She underwent a left lumpectomy with pathology confirming triple-negative invasive ductal carcinoma with associated high-grade DCIS and margins negative for both invasive and in situ disease, staining positive for mammaglobin. There was no suspicion that this was a colon metastasis. She elected to omit sentinel lymph node biopsy, radiation, and breast-specific chemotherapy.

The final pathology of the back mass continued to be a diagnostic dilemma. To further characterize the origin, the histologic sections from the back and colon specimens were sent for an expert opinion at another institution. A panel of newly performed immunostains showed the neoplastic cells to be positive for CK20 and negative for CDX2, CK7, p63, GATA3, and mammaglobin, which is compatible with a metastasis from colonic primary. The patient was discussed at our multidisciplinary tumor board. We recommended a colon-specific chemotherapy regimen, which the patient declined. She returned to the clinic 4 months after the initial back mass excision and reported pruritus of her right back along the incision line with an associated lump. She also noted a lump in her right axilla, which was painful with arm movement. Exam revealed a well-healed right upper back scar with a 1.5 × 1.5 cm raised flesh-colored nodule with bluish discoloration proximal to the scar at the midpoint, as well as a well-healed right axillary scar with a firm palpable nodule 5 cm inferior to the scar. The patient was offered a wide resection in the OR, but due to the severe pruritic nature of this lesion, she preferred narrow excision in the office for immediate palliation and diagnosis. Pathology revealed this tissue was morphologically identical to her previous skin lesion, confirming a metastatic poorly differentiated carcinoma of colon primary. Caris testing was performed, and there were no actionable mutations.

The patient represented to medical attention one month later with a large bowel obstruction. Imaging showed recurrence at the colonic anastomosis, and this was managed nonoperatively. At this point, the patient agreed to palliative chemotherapy. She completed one cycle of capecitabine and oxaliplatin but tolerated this regimen poorly and was readmitted to the hospital twice. Subsequently, she declined any further treatment and went home on hospice.

## 3. Discussion

At the time of diagnosis, distant metastases are present in approximately 18% of all colorectal cancer cases with 95% of these patients having either liver, lung, bone, or brain lesions [14]. Cutaneous metastases from an internal primary malignancy are uncommon and are a particularly rare manifestation of colon adenocarcinoma. Approximately 4–6% of metastatic colon cancers are associated with cutaneous lesions, which are primarily found on the abdominal skin at the site of postoperative incisional scars likely due to direct seeding [15]. The average time of presentation with skin metastases following a curative operation for CRC is about 2 years, but monitoring for these lesions should begin postoperatively as their presence can be the first detectable sign of disease progression [16].

The morphologic presentation of skin metastases varies widely and seemingly independent of the underlying primary malignancy. Lesions can present as nodules, papules, plaques, masses, or ulcers. Atypical presentations can include alopecia plaques or lesions mimicking morphea, follicular or sebaceous cysts, dermatofibromas, pyogenic granulomas, hemangiomas, herpes zoster eruption, cellulitis, or erysipelas [17]. This variable presentation often leads to a missed or delayed diagnosis. Therefore, providers must practice a high degree of clinical suspicion when investigating any new skin lesion in a patient with a recent or remote history of malignancy, even if there were no sites of distant metastases at initial diagnosis. Nevertheless, given this large variation in the visual appearance of these lesions, a biopsy and pathologic analysis are crucial in making the diagnosis.

Histologically, cutaneous malignant lesions are broadly classified as adenocarcinoma, squamous carcinoma, undifferentiated carcinoma, and other types. However, the morphology of metastatic deposits alone is typically not enough to establish the primary tumor source [18]. This is in part due to the fact that differentiation grade of metastatic tumors can change and obscure typical histologic features [19]. For this reason, the diagnosis of cutaneous metastatic carcinoma depends heavily on immunohistochemical stains to confirm the diagnosis. IHC markers of lower gastrointestinal origin identified in our case included high-molecular weight keratin, BerEP4, and CK20. CK20 is particularly useful in differentiating adenocarcinomas originating from the gastrointestinal tract from nongastrointestinal adenocarcinoma [20].

In general, the median survival after the diagnosis of cutaneous metastases is 6.5 months, with CRC cases of cutaneous lesion survival falling short of that at 4.4 months [21]. This is in comparison to all cases of CRC with distant metastases in which the median survival is between 9 and 19 months depending on whether treatment consists of best supportive care or combination chemotherapy [22]. This clear discrepancy in the length of survival may be due to the fact that cutaneous lesions are rarely the sole site of metastasis in these patients and are typically a sign of advanced disease. In fact, cutaneous metastases usually occur only with concomitant hepatic involvement [8–12]. However, if the skin is the only site of distant metastasis, as in the case of our patient, this may be a more favorable presentation.

While there are no specific guidelines for the management of cutaneous metastases in CRC, it is reasonable to proceed with the standard treatment for any CRC with distant metastases, systemic chemotherapy or targeted therapy, depending on mutational analysis, or a clinical trial. If the cutaneous lesion is the sole site of metastasis, wide local excision should be considered as surgical resection in cases of metastatic CRC is associated with superior outcomes for long-term survival [23].

Cutaneous lesions are an exceedingly rare site of metastasis for colon adenocarcinoma, and their clinical presentation can vary widely. Therefore, it is important for providers to investigate any new skin lesion in a patient with a recent or remote history of malignancy, even if there were no sites of distant metastasis at initial diagnosis. Molecular staining and pathological comparison to the primary tumor, when possible, can aid in the diagnosis of a metastatic skin lesion. Treatment options should include wide local excision if it is a solitary lesion or systemic chemotherapy if there are multiple sites of metastases.

## Data Availability

The patient data used to support the findings of this study are included within the article.
